# Comparing survival curves based on medians

**DOI:** 10.1186/s12874-016-0133-3

**Published:** 2016-03-16

**Authors:** Zhongxue Chen, Guoyi Zhang

**Affiliations:** Department of Epidemiology and Biostatistics, School of Public Health, Indiana University Bloomington, 1025 E. 7th street, SPH C104, Bloomington, IN 47405 USA; Department of Mathematics and Statistics, University of New Mexico, Albuquerque, NM 87131 USA

**Keywords:** Censored data, Cochran test, Nonparametric test, Survival analysis

## Abstract

**Background:**

Although some nonparametric methods have been proposed in the literature to test for the equality of median survival times for censored data in medical research, in general they have inflated type I error rates, which make their use limited in practice, especially when the sample sizes are small.

**Methods:**

In this paper, we propose a new nonparametric test with a simple test statistic to compare median survival times.

**Results:**

The results from our comprehensive simulation study show the new test controls type I error rate very well under all situations considered, even for small sample sizes. In addition, it has comparable detecting power compared to existing methods. Another advantage of the proposed test is its relatively simple formula that requires less computation.

**Conclusions:**

We propose a new statistical method for comparing survival curves based on their medians. The new method can be easily implemented and applied to censored event time data.

## Background

In analyzing data with censored observations, it is a common task to compare several survival curves. Many nonparametric tests based on the Kaplan-Meier estimator for survival curves [1], such as Gehan’s generalized Wilcoxon test, the Cox-Mantel test, the logrank test, and Peto tests, have been proposed and implemented in major statistical software [2]. In addition, Efron proposed a test based on the conditional probability of censored data for two samples cases [3, 4].

Due to the skewness of survival data, the median survival time is thought to be an important measurement of the survival distribution. If two crossing survival curves [5] are different but their median survival times are similar, then comparing the survival medians or quantiles rather than the curves is more appropriate to answer some research questions. Several nonparametric tests for comparing median survival times have been proposed in the literature [6–11]. Brookmeyer and Crowley proposed a test (called BC test hereafter) based on the weighted survival curve and the weighted median [11]. In their test, the individual survival curve for each group and their weighted survival median time need to be estimated by using Kaplan-Meier estimator; then the survival probabilities from all groups at the time point of the estimated weighted survival median are compared. The authors showed that the test statistic has an asymptotic chi-square distribution with degrees of freedom equal to the number of groups minus one, under the assumption that all of the groups have the same survival distribution. In order to get this test statistic, we need to estimate a covariance matrix and calculate its generalized inverse, which may be a tedious task [8]. Another limitation of the BC test is its inflated type I error rate, which may cause serious problems when sample sizes are small [9].

Rahbar et al. proposed another nonparametric test (called RC test hereafter) which is directly based on the estimated median survival times [9]. Although some methods of estimating the median survival time, such as Reid’s method [12], have been proposed, we found that RC method using those approaches to estimate the variances had poor performance. Alternatively, resampling-based methods, such as bootstrap can be used to estimate the variances. Through simulation study, the authors showed that the RC test performs similarly to the BC test when sample sizes are large. However, when sample sizes are medium (e.g., 50 per group), although the RC test has empirical type I error rates less than the BC test, they are still inflated.

Motivated by avoiding calculating the inverse matrix in the BC test statistic, recently Tang and Jeong proposed a simple nonparametric test (called TJ test hereafter) based on the estimated conditional probability of the censored time which is greater than the overall median [4, 8]. Their test statistic is a weighted sum of statistics obtained from a series of contingency tables. The authors claimed that their test statistic under the null hypothesis could be approximated by a chi-square distribution [8].

In this paper, we propose a new nonparametric test with a simple formula. Through comprehensive simulation, we show that the proposed test controls type I error rate very well, even for situations where sample sizes are very small. Our simulation results also show that the existing methods have inflated type I error rates, which is very serious when sample sizes are small. We also illustrate the use of the proposed test by a real data application.

## Method

Denoted by ${X}_{i1},{X}_{i2},\dots, {X}_{i{n}_i}$ (i =1, 2,…, K) the *n*_*i*_ independent random survival times from the *i*^th^ group. For right censored data, since some of the survival times may not be observed completely, the data are recorded in the form (*Z*_*ij*_, *δ*_*ij*_), *j* = 1, 2, …, *n*_*i*_, i = 1,2,…, K, where *Z*_*ij*_ = *Min*(*X*_*ij*_, *Y*_*ij*_), *δ*_*ij*_ = *I*(*X*_*ij*_ ≤ *Y*_*ij*_), *Y*_*ij*_ is the censoring time of the *j*^th^ subject in the *i*^th^ group, and *I* (⋅) is the indicator function. In this paper, we assume the generic random variables *X* and *Y* are independent with survival distributions *F*_*i*_(*x*) = *P*(*X*_*ij*_ > *x*), *G*_*i*_(*y*) = *P*(*Y*_*ij*_ > *y*), respectively. The median survival time of F_i_ is defined as *θ*_*i*_ = inf(*t* : *F*_*i*_(*t*) ≤ 0.5). It can be estimated by $\widehat{\theta_i}= \inf \kern0.3em \left(t:\widehat{F_i}(t)\le 0.5\right)$, where $\widehat{F_i}$ is the Kaplan -Meier estimator of *F*_*i*_ [1].

The proposed test statistic is as follows:

1 $$ C={\displaystyle {\sum}_{i=1}^K{w}_i}{\left({\eta}_i-{\displaystyle {\sum}_{j=1}^K{h}_j}\kern0.5em {\eta}_j\right)}^2, $$

where ${\eta}_i=\widehat{F_i}\left({\widehat{\theta}}_0\right)$, ${\widehat{\theta}}_0$ is the overall sample median survival time obtained from the pooled data set, ${w}_i=1/{\widehat{\upsigma}}_{\mathrm{i}}^2$, ${\widehat{\upsigma}}_{\mathrm{i}}^2$ is a consistent estimate for variance of ${\eta}_i$, and *h*_*i*_ = *w*_*i*_/∑_*j* = 1_^*K*^*w*_*j*_.

For the test statistic *C* in (1), we have the following theorem:

**Theorem 1**. Under the null hypothesis that the K survival curves have the same median, statistic *C* has an asymptotic chi-square distribution with K-1 df.

Proof: It is known that under the null hypothesis, ${\eta}_i$ has an asymptotic normal distribution *N*(0.5, *σ*_*i*_^2^) [11]. Then from Theorem 1 of Chen et. al [13] and Slutsky’s theorem, ${\displaystyle {\sum}_{i=1}^K{w}_i}{\left({\eta}_i-{\displaystyle {\sum}_{j=1}^K{h}_j}\kern0.5em {\eta}_j\right)}^2$ has an asymptotic chi-square distribution with df = K-1 given ${\widehat{\upsigma}}_{\mathrm{i}}^2$ is a consistent estimate for the variance of ${\eta}_i$.

### Variance estimate for ${\eta}_i$

Although the Greenwood method [14] can be used to estimate the variance of ${\widehat{F}}_i\left({\widehat{\theta}}_0\right)$, in general, the Greenwood approach tends to underestimate the variance [15]. In fact, it is well known that *V*(*Y*) = *E*[*V*(*Y*|*X*)] + *V*[*E*(*Y*|*X*)]. With Y as the estimated survival function and X as the survival data, the variance decomposition *V*(*Y*) = *E*[*V*(*Y*|*X*)] + *V*[*E*(*Y*|*X*)] can be applied. The Greenwood estimate is an approximation of *E*[*V*(*Y*|*X*)]. Therefore if we ignore the second part, *V*[*E*(*Y*|*X*)], the Greenwood estimate will tend to underestimate the variance. It is also known that a Cochran-type test statistic such as *C* in (1) is anti-conservative [13] and is an increasing function of the weights *w*_*i*_ [16]. Therefore, if we replace ${\widehat{\upsigma}}_{\mathrm{i}}^2$ in *C* by the Greenwood estimate, the type I error rate will become inflated [13].

For the first part, *E*[*V*(*Y*|*X*)], we will use the Greenwood estimate; the second part, *V*[*E*(*Y*|*X*)], can be estimated using bootstrap. However, here we propose a simple approximation to it: for group *i*, we estimate the median survival time ${\widehat{\theta}}_i$ using the Kaplan-Meier method and find the uncensored time ${\widehat{\theta}}_{i1}$ which is the closest one to ${\widehat{\theta}}_i$, then we obtain the variance between ${\widehat{F}}_i\left({\widehat{\theta}}_i\right)$ and ${\widehat{F}}_i\left({\widehat{\theta}}_{i1}\right)$, ${\left[{\widehat{F}}_i\left({\widehat{\theta}}_i\right)-{\widehat{F}}_i\left({\widehat{\theta}}_{i1}\right)\right]}^2/2$. This approximation works well because either of the two times will be close to the median from the bootstrap. It is easily seen that this estimated variance has an order of *n*_*i*_^− 2^. Hence, for large sample sizes, this part can be ignored and the new test statistic will have a similar value as the BC test statistic. Based on the simulation results (see below), when sample sizes are greater than 100 (as in Tables 1 and 2), BC and the proposed test have similar empirical type I error rates, indicating that under those situations it is safe to ignore the second part. Nevertheless, when sample sizes are smaller than 30 (see Tables 4 and 5), the second part should not be ignored, as it will result in serious inflated type I error rates (see the Results section).

**Table 1:** The empirical type I error rates of the tests at the significance level of 0.05 when the survival times have the same uniform, exponential, or log-normal distributions with sample sizes *n*_1_ = 100, *n*_2_ = 150, *n*_3_ = 150, *n*_4_ = 200 (simulation study with 10,000 replicates for each situation)

Distributions and parameters	Censoring rate	BC	TJ	New
Uniform distribution *θ* = (10,10,10,10) *a* = (4,4,4,4) *c* = (2,2,2,2)	0	0.053	0.049	0.051
	0.1	0.052	0.070	0.047
	0.2	0.057	0.092	0.049
	0.3	0.057	0.114	0.047
Exponential distribution *θ* = (10,10,10,10) *a* = (0.1,0.1,0.1,0.1)	0	0.050	0.047	0.047
	0.1	0.052	0.056	0.050
	0.2	0.053	0.068	0.050
	0.3	0.051	0.083	0.047
Log-normal distribution *θ* = (log(10), log(10), log(10), log(10)) *a* = (0.3,0.3,0.3,0.3)	0	0.057	0.052	0.054
	0.1	0.055	0.065	0.050
	0.2	0.054	0.082	0.050
	0.3	0.054	0.104	0.050

**Table 2:** The empirical type I error rates of the tests at the significance level 0.05 when the survival times have uniform, exponential, log-normal,uniform and exponential, uniform and log-normal, or exponential and log-normal distributions with equal medians but unequal variances and sample sizes *n*_1_ = 100, *n*_2_ = 150, *n*_3_ = 150, *n*_4_ = 200

Distributions and parameters	Censoring rate	BC	TJ	New
Uniform distribution *θ* = (10,10,10,10) *a* = (4,4,4,4) *c* = (2,3,4,5)	0	0.061	0.057	0.058
	0.1	0.057	0.068	0.053
	0.2	0.061	0.089	0.054
	0.3	0.062	0.109	0.053
Exponential distribution *θ* = (10-2log(2),10-3log(2), 10-4log(2),10-5log(2)) *a* = (1/2,1/3,1/4,1/5)	0	0.060	0.057	0.058
	0.1	0.067	0.071	0.062
	0.2	0.062	0.079	0.057
	0.3	0.064	0.098	0.057
Log-normal distribution *θ* = (log(10), log(10), log(10), log(10)) *a* = (0.2, 0.3, 0.4, 0.5)	0	0.057	0.053	0.055
	0.1	0.063	0.073	0.059
	0.2	0.064	0.094	0.058
	0.3	0.060	0.117	0.054
Two uniform and two exponential distributions *θ* = (10, 10, 10-10log(2),10-10log(2)) *a* = (0.1, 0.1, 0.1, 0.1) *c* = (0.01, 0.01, 0.01, 0.01)	0	0.055	0.050	0.052
	0.1	0.053	0.080	0.047
	0.2	0.055	0.102	0.049
	0.3	0.059	0.118	0.051
Two uniform and two log-normal distributions *θ* = (10,10, log(10), log(10)) *a* = (5,5,0.3,0.3) *c* = (2,2,2,2)	0	0.058	0.081	0.054
	0.1	0.063	0.087	0.057
	0.2	0.061	0.107	0.054
	0.3	0.055	0.123	0.048
Two log-normal and two exponential distributions *θ* = (log(10), log(10),10-log(2)/0.3,10-log(2)/0.3) *a* = (0.3,0.3,0.3,0.3)	0	0.052	0.049	0.049
	0.1	0.050	0.058	0.047
	0.2	0.058	0.082	0.054
	0.3	0.056	0.095	0.050

## Results

### Simulation study

To assess the performance of the proposed test, we conducted a comprehensive simulation study to compare it with the BC test and TJ test. RC is excluded in the simulation study since it has similar performance as the BC test in general, but has worse performance when sample sizes are small [6]. All of the simulation were conducted by using R, with the R package “survival” [17].

In this simulation study, the alternative hypothesis is the medians are not the same among several populations. We choose K = 4 groups and considered the following survival time distributions (F_i_)): (a) all uniform, U(−*a*_*i*_/*c*_*i*_, *a*_*i*_/*c*_*i*_) + *θ*_*i*_, (b) all exponential, exp(*a*_*i*_) + *θ*_*i*_, (c) all log-normal, lnorm(*θ*_*i*_, a_*i*_), (d) two uniform and two exponential, (e) two uniform and two log-normal, and (f) two exponential and two log-normal. The censoring time distributions (G_i_) are uniform U(−*a*_*i*_/*c*_*i*_, *a*_*i*_/*c*_*i*_ + (1 − 2*p*_*i*_)/*p*_*i*_) + *θ*_*i*_, exponential exp(*a*_*i*_*p*_*i*_/(1 − *p*_*i*_)) + *θ*_*i*_, and log-uniform lunif[*θ*_*i*_ + *a*_*i*_*U*(−2, − 2 + 2/p_*i*_)], respectively, when the survival time distributions are uniform, exponential, and log-normal. Here p_*i*_ is the censoring rate, which is set to be 0 (noncensoring), 0.1, 0.2, and 0.3 in the simulation. The type I error rates and power values are estimated by the empirical rejection proportions from 10,000 replicates at preset significance level 0.05. We compare these tests under both large sample sizes (*n*_1_ = 100, *n*_2_ = 150, *n*_3_ = 150, *n*_4_ = 200) and small sample sizes (*n*_1_ = 20, *n*_2_ = 25, *n*_3_ = 25, *n*_4_ = 30) situations.

Tables 1, 2 and 3 are the simulation results under the situations of large sample sizes. Table 1 reports the empirical type I error rates for these methods when all of the four groups have the same survival time distribution and the same censoring time distribution. It shows that the BC test and the proposed test control type I error rate very close to the nominal level. The TJ test can control type I error rate only when the censoring rate is 0. With the censoring rate increases, the TJ test has larger inflated empirical type I error rates. The empirical type I error rate can be as large as 0.114, for example, when the survival time distribution is uniform and the censoring rate is 0.3.

**Table 3:** The empirical powers of the tests at the significance level 0.05 when the survival times have uniform, exponential, log-normal,uniform and exponential, uniform and log-normal, or exponential and log-normal distributions with unequal medians and sample sizes *n*_1_ = 100, *n*_2_ = 150, *n*_3_ = 150, *n*_4_ = 200

Distributions and parameters	Censoring rate	BC	TJ	New
Uniform distribution *θ* = (10,10,10.5,10) *a* = (4,4,4,4) *c* = (2,2,2,2)	0	0.602	0.587	0.594
	0.1	0.563	0.606	0.549
	0.2	0.537	0.622	0.518
	0.3	0.509	0.635	0.490
Exponential distribution *θ* = (10,10,10,10) *a* = (0.1,0.15,0.15,0.1)	0	0.837	0.829	0.833
	0.1	0.822	0.831	0.818
	0.2	0.799	0.830	0.793
	0.3	0.765	0.826	0.756
Log-normal distribution *θ* = (10,14,14,10) *a* = (1,1,1,1)	0	0.804	0.794	0.797
	0.1	0.776	0.800	0.769
	0.2	0.729	0.787	0.718
	0.3	0.696	0.798	0.681
Two uniform and two exponential distributions *θ* = (10,13,13-10log(2),10-10log(2)) *a* = (0.1,0.1,0.1,0.1) *c* = (0.01,0.01,0.01,0.01)	0	0.889	0.883	0.884
	0.1	0.838	0.875	0.827
	0.2	0.817	0.873	0.801
	0.3	0.798	0.874	0.779
Two uniform and two log-normal distributions *θ* = (10,12,2.5,2.5) *a* = (1,1,1,1) *c* = (0.2,0.2,0.2,0.2)	0	0.847	0.821	0.845
	0.1	0.751	0.813	0.766
	0.2	0.695	0.813	0.706
	0.3	0.651	0.815	0.644
Two log-normal and two exponential distributions *θ* = (log(10),log(13),13-10log(2),10-10log(2)) *a* = (1, 1, 0.1, 0.1)	0	0.799	0.790	0.793
	0.1	0.761	0.776	0.752
	0.2	0.744	0.788	0.727
	0.3	0.718	0.795	0.699

Table 2 gives the empirical type I error rates for each method when the four survival distributions have the same medians but with different variances. Once again, the BC test and the new test have empirical type I error rates close to the nominal level 0.05. On the other hand, the TJ test still has inflated sizes when there are censored data present.

Table 3 shows the empirical powers for each method when the four median survival times are not the same (i.e., the null hypothesis is not true). In general, the BC test and the proposed test have close power values. When there are censored data, the TJ test obtains larger power values. This may be because this test has inflated type I error rates.

Tables 4, 5 and 6 report the simulation results for small sample sizes (*n*_1_ = 20, *n*_2_ = 25, *n*_3_ = 25, *n*_4_ = 30) and different levels of censoring. In Table 4 where the null hypothesis is true and the survival time and censoring time each has the same distribution for the four groups, the proposed test controls type I error rate well. Unlike its performance under large sample size situations, when sample sizes are small, the BC test also has inflated type I error rates even for noncensored data. Once again the TJ test cannot control the type I error rate when the censoring rate is not 0.

**Table 4:** The empirical type I error rates of the tests at the significance level 0.05 when the survival times have the same uniform, exponential, or log-normal distributions with sample sizes *n*_1_ = 20, *n*_2_ = 25, *n*_3_ = 25, *n*_4_ = 30

Distributions and parameters	Censoring rate	BC	TJ	New
Uniform distribution *θ* = (10,10,10,10) *a* = (4,4,4,4) *c* = (2,2,2,2)	0	0.071	0.051	0.054
	0.1	0.080	0.073	0.053
	0.2	0.082	0.092	0.045
	0.3	0.090	0.121	0.045
Exponential distribution *θ* = (10,10,10,10) *a* = (0.1,0.1,0.1,0.1)	0	0.066	0.046	0.049
	0.1	0.074	0.055	0.056
	0.2	0.078	0.068	0.051
	0.3	0.084	0.087	0.050
Log-normal distribution *θ* = (log(10), log(10), log(10), log(10)) *a* = (0.3,0.3,0.3,0.3)	0	0.068	0.048	0.053
	0.1	0.073	0.064	0.051
	0.2	0.081	0.085	0.052
	0.3	0.080	0.103	0.044

**Table 5:** The empirical type I error rates of the tests at the significance level 0.05 when the survival times have uniform, exponential, log-normal,uniform and exponential, uniform and log-normal, or exponential and log-normal distributions with equal medians but unequal variances and sample sizes *n*_1_ = 20, *n*_2_ = 25, *n*_3_ = 25, *n*_4_ = 30

Distributions and parameters	Censoring rate	BC	TJ	New
Uniform distribution *θ* = (10,10,10,10) *a* = (4,4,4,4) *c* = (2,3,4,5)	0	0.087	0.062	0.067
	0.1	0.087	0.075	0.057
	0.2	0.087	0.086	0.051
	0.3	0.099	0.118	0.053
Exponential distribution *θ* = (10-2log(2),10-3log(2),10-4log(2),10-5log(2)) *a* = (1/2,1/3,1/4,1/5)	0	0.084	0.062	0.064
	0.1	0.086	0.067	0.060
	0.2	0.089	0.078	0.062
	0.3	0.100	0.100	0.054
Log-normal distribution *θ* = (log(10), log(10), log(10), log(10)) *a* = (2,3,4,5)/10	0	0.078	0.057	0.060
	0.1	0.088	0.077	0.064
	0.2	0.090	0.093	0.063
	0.3	0.087	0.109	0.052
Two uniform and two exponential distributions *θ* = (10, 10, 10-10*log(2),10-10*log(2)) *a* = (0.1,0.1,0.1,0.1) *c* = (0.01,0.01,0.01,0.01)	0	0.074	0.052	0.058
	0.1	0.088	0.089	0.049
	0.2	0.093	0.106	0.046
	0.3	0.092	0.119	0.039
Two uniform and two log-normal distributions *θ* = (10,10, log(10), log(10)) *a* = (5,5,0.3,0.3) *c* = (2,2,2,2)	0	0.076	0.056	0.058
	0.1	0.085	0.083	0.050
	0.2	0.088	0.105	0.049
	0.3	0.097	0.132	0.047
Two log-normal and two exponential distributions *θ* = (log(10), log(10),10-log(2)/0.3,10-log(2)/0.3) *a* = (0.3,0.3,0.3,0.3)	0	0.068	0.049	0.051
	0.1	0.078	0.062	0.055
	0.2	0.083	0.080	0.054
	0.3	0.096	0.111	0.059

**Table 6:** The empirical powers of the tests at the significance level 0.05 when the survival times have uniform, exponential, log-normal,uniform and exponential, uniform and log-normal, or exponential and log-normal distributions with unequal medians and sample sizes *n*_1_ = 20, *n*_2_ = 25, *n*_3_ = 25, *n*_4_ = 30

Distributions and parameters	Censoring rate	BC	TJ	New
Uniform distribution *θ* = (10,10,10.5,10) *a* = (4,4,4,4) *c* = (2,2,2,2)	0	0.169	0.133	0.140
	0.1	0.171	0.153	0.126
	0.2	0.175	0.186	0.117
	0.3	0.177	0.216	0.104
Exponential distribution *θ* = (10,10,10,10) *a* = (0.1,0.15,0.15,0.1)	0	0.229	0.184	0.196
	0.1	0.233	0.199	0.201
	0.2	0.233	0.218	0.187
	0.3	0.221	0.231	0.160
Log-normal distribution *θ* = (log(10), log(14), log(14), log(10)) *a* = (1,1,1,1)	0	0.231	0.185	0.201
	0.1	0.212	0.190	0.169
	0.2	0.210	0.218	0.163
	0.3	0.201	0.240	0.140
Two uniform and two exponential distributions *θ* = (10,13,13-10log(2),10-10log(2)) *a* = (0.1,0.1,0.1,0.1) *c* = (0.01,0.01,0.01,0.01)	0	0.277	0.229	0.234
	0.1	0.266	0.270	0.190
	0.2	0.264	0.295	0.172
	0.3	0.258	0.315	0.158
Two uniform and two log-normal distributions *θ* = (10,12,2.5,2.5) *a* = (1,1,1,1) *c* = (0.2,0.2,0.2,0.2)	0	0.288	0.218	0.275
	0.1	0.250	0.257	0.240
	0.2	0.249	0.294	0.218
	0.3	0.253	0.329	0.197
Two log-normal and two exponential distributions *θ* = (log(10),log(13),13-10log(2),10-10log(2)), *a* = (1,1,0.1,0.1)	0	0.229	0.183	0.182
	0.1	0.223	0.195	0.174
	0.2	0.228	0.217	0.168
	0.3	0.220	0.242	0.147

When the four survival distributions have the same median with different variances, the empirical type I error rates of these tests are given in Table 5. If there is no censored data, the TJ test has empirical type I error rates close to the nominal level; but the BC test still has inflated type I error rates. When there are censored data, both the BC test and the TJ test have inflated type I error rates. In contrast, the proposed test can control type I error rate quite well under all situations considered.

Table 6 reports the empirical powers for the three methods when the null hypothesis is not true. In general, the empirical powers from the new test are smaller than those from the BC test and the TJ test. When the censoring rate is high (e.g., 0.3), the power values for the TJ test are higher than that from the BC test. This can be explained by their performance of controlling type I error rates as seen in Tables 4 and 5; both the BC test and the TJ test have inflated type I error rates for most of the situations considered in this simulation study when sample sizes are small.

### Real data application

To illustrate the use of the proposed test, we conduct a real data application. In a study to investigate the relationship between diet and the development of tumors, 90 rats were divided into three groups and 30 rats in each group were fed with the same low-fat, saturated fat or unsaturated fat. The rats had the same age, species and similar physical condition. The rats were observed for 200 days, and the times to develop a tumor were recorded. This data set is available from Table 3.4 of Lee and Wang’s book [2]. The estimated median survival times (tumor-free time) were 191, 107, and 91 days for the low-fat, saturated fat and unsaturated fat groups, respectively.

The survival curves (not shown) estimated by the Kaplan-Meier method for the three diet groups indicate the expected tumor-free time for rats fed with low-fat is longer than that for the rats fed with saturated fat, which in turn is longer than that for the rats fed with unsaturated fat. We compare the median survival times for these three groups using the BC test, TJ test and the proposed test. The *p*-values from the three tests are 0.0059, 0.017, and 0.0043, respectively. All of the three methods reject the null hypothesis at significance level 0.05. In addition, when testing for the equality of survival curves, the *p*-values from the log-rank test and Wilcoxon test are 3.14 × 10^-5^ and 0.0020, respectively.

## Discussion and conclusion

Although several nonparametric tests have been proposed to compare the median survival times, they are anti-conservative which make their application limited, especially for the situations where the sample sizes are small. To overcome this shortcoming, in this paper, we proposed a new nonparametric approach to testing for the equality of median survival times. Our comprehensive simulation study shows that, unlike existing methods, the new test controls type I error rate very well under all situations considered, even if the sample sizes are small. On the other hand, the detecting power of this new method is comparable to BC when both can control type I error rate (i.e., large sample size). TJ method usually has larger power values; however, this mainly because it has inflated type I error rate, even for large sample sizes (see Tables 1 and 2). The BC test has similar power values as the proposed test when sample sizes are large; but it has inflated type I error rate when sample sizes are small. Therefore, the proposed test is preferred over the BC test.

The new test statistic has a very simple formula; it neither requires calculating a matrix and its inverse, nor relies on the time consuming resampling-based methods, such as bootstrap. Therefore, unlike those re-sampling based methods, this test needs less computation and can be easily implemented. Furthermore, although the proposed test is designed for detecting the difference of the median survival times, it can be extended without any difficulty to compare survival quantiles. In addition, the proposed test is also suitable for uncensored data.
